# Knockdown of circ_0001679 alleviates lipopolysaccharide-induced MLE-12 lung cell injury by regulating the miR-338-3p/ mitogen-activated protein kinase 1 axis

**DOI:** 10.1080/21655979.2022.2034564

**Published:** 2022-03-10

**Authors:** Shenggui Lu, Xinmiao Wu, Shuai Xin, Jing Zhang, Hanying Lin, Yu Miao, Yixin Li

**Affiliations:** a Intensive Care Unit, The 910th Hospital of People’s Liberation Army Joint Logistic Support Force, Quanzhou, China; b Department of Emergency, Hospital of Traditional Chinese Medicine, Quanzhou, Fujian Provice, China; c Department of Anesthesiology, The 910th Hospital of People’s Liberation Army Joint Logistic Support Force, China; d Department of Nursing, The 910th Hospital of People’s Liberation Army Joint Logistic Support Force, Quanzhou, Fujian, China

**Keywords:** Septic acute lung injury, circ_0001679, miR-338-3p, MAPK1

## Abstract

The upregulation of circ_0001679 was reported in lipopolysaccharide (LPS)-induced lung injury mouse model, but its functional roles and mechanisms in LPS-induced lung injury remain to be investigated. In this study, we aimed to explore the potential role of circ_0001679 in septic acute lung injury. We initially established an *in vitro* lung cell injury model using LPS-treated MLE-12 cells. siRNAs targeting circRNA_0001679 were employed to stably knock down circRNA_0001679, followed by functional assays to investigate the effect of circRNA_0001679 silencing. The levels of inflammatory cytokines such as IL-6, IL-β and TNF-α (Tumor necrosis factor-α) were detected by ELISA (Enzyme-linked immunosorbent assay). Meanwhile, protein levels of Bcl-2, cleaved-caspase 3, Bax, and MAPK1 (Mitogen-Activated Protein Kinase 1) proteins expression level were measured by Western blot. We found that Circ_0001679 was upregulated in LPS-induced MLE-12 cells, and silencing circ_0001679 attenuated the growth inhibition and suppressed apoptosis induced by LPS. Circ_0001679 knockdown also lowered levels of IL-6, IL-β and TNF-α, and prevent the activation of cleaved-caspase 3 protein. We further revealed that circ_0001679 functioned as a sponge of miR-338-3p to negatively regulate miR-338-3p activity. miR-338-3p downregulated its downstream target MAPK1, while the upregulation of circ_0001679 maintained a high-level expression of MAPK1 by suppressing miR-338-3p. Collectively, our study indicates that circ_0001679/miR-338-3p/MAPK1 axis may play an important role in the pathogenesis of acute lung injury (ALI).

## Graphical abstract

**Figure:**
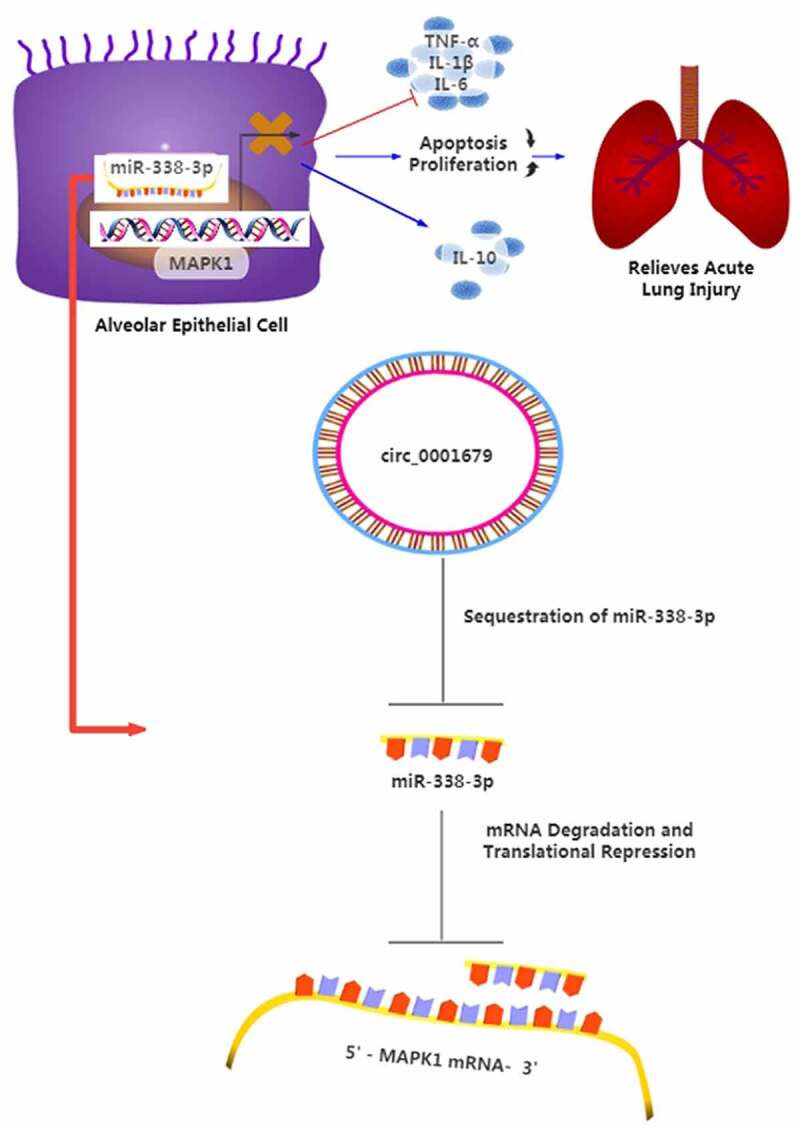

## Introduction

A cute respiratory distress syndrome (ARDS)/acute lung injury (ALI) is characterized by non-cardiogenic pulmonary edema, acute respiratory distress, as well as persistent inflammatory response caused by various pulmonary and external factors [1]. ALI/ARDS treatment modalities include general supportive care and protective mechanical ventilation [2]. Although these treatments have improved the survival rate of ALI/ARDS patients, the therapeutic outcome has not met expectations with a mortality rate between 40% and 46% [3]. Therefore, understanding the molecular mechanisms underlying the pathogenesis of acute lung injury is crucial for formulating novel therapeutic approaches.

The main pathophysiological features of ALI/ARDS include the migration of inflammatory cells, fibroproliferation, and activation of apoptosis in alveolar epithelial cells [4]. Inflammatory responses have been recognized as a key factor in ALI pathophysiology and a self-limiting inflammatory response is crucial for regulating the progression of ALI [5]. In the early stage of ALI, local neutrophils and macrophages infiltrate in the lung tissues and secrete various cytokines like TNF-α, IL-6, and IL-1β, which can further activate leukocytes and cause systemic inflammatory response syndrome (SIRS) if inflammation is not controlled [6,7]. Inflammatory responses and cytokines therefore are key contributors to the development and pathogenic progression of ALI/ARDS [8]. During the development of ALI/ARDS, alveolar epithelial cells (AEC) are the main target cells that suffer from cell damage and apoptosis due to inflammations [9]. Apoptosis can be triggered by external pathways or internal pathways originating from the mitochondria. Both pathways can eventually activate cysteine aspartate-specific proteases (caspases), which function as the executioner proteases inducing morphological and biochemical changes during apoptosis [10]. After the onset of diffuse alveolar injury, DNA damage level is increasing and there is an elevated expression of pro-apoptotic protein Bax in Alveolar epithelial cell (AEC) [11]. In the treatment of ALI/ARDS, interventions to control the ‘waterfall’ effect of inflammatory responses and ‘cytokine storms’ in the lung are considered as key measures to prevent alveolar epithelial cell apoptosis [12].

The etiology of ALI is complicated, including sepsis, pneumonia, gastric content aspiration, severe trauma, acute pancreatitis, and blood transfusion [13]. Among these pathogenic factors, sepsis is the most common cause of ALI/ARDS [14]. Lipopolysaccharide (LPS) is the main component of gram-negative bacterial cell wall that stimulates inflammatory cells and activates multiple pathways like JAK/STAT, MAPK, or NF-κB to induce inflammatory cytokines [15–17]. LPS has been recognized as an important factor in ALI-related inflammatory reaction, which is widely used for inducing acute lung injury in animal and cell models [18,19]. Circular RNAs (circRNAs) have been recently implicated in sepsis-related lung injury and inflammatory responses [20,21]. CircRNAs are non-coding RNAs with a covalent closed-loop structure, which are highly stable and show cell-type specific expression. They can not only sponge RNA-binding proteins (RBPs) and microRNAs (miRNAs), but also modulate the translation and transcription of genes involved in a wide range of biological processes [20,21]. MicroRNAs (miRNAs) are small endogenous non-coding RNAs (19–23 nucleotides) which can modulate post-transcriptional gene expression [22]. miRNAs have been found to regulate numerous cellular processes, including cell growth, invasion, apoptosis, differentiation, inflammatory response, immune function and tumorigenesis [23,24]. In sepsis-induced lung injury, circRNAs have been reported to modulate inflammatory responses by sponging specific miRNAs [25,26]. Circ_0001679 was found to be highly expressed in a mice model of ALI, and P2X7R antagonist ameliorates lung injury by regulating the expression of circ_0001212 and circ_0001679 [21]. In human bronchial epithelial cells, it has been reported that circ_0038467 is able to regulate LPS-induced inflammatory injury by sponging miR-338-3p [26].

In our exploratory study, we found that circ_0001679 was upregulated in LPS-induced MLE-12 cells, we therefore hypothesized that circ_0001679 plays a functional role in LPS-induced cell damages in MLE-12 cells. siRNAs targeting circRNA_0001679 were employed to stably knock down circRNA_0001679 and we investigated the cell damages and inflammatory responses after circRNA_0001679 silencing. We further identified miR-338-3p as a downstream miRNA target of circRNA_0001679, and we performed functional assays to demonstrate that circ_0001679/miR-338-3p/ MAPK1 axis may play an important role in the cell model of ALI.

## Materials and methods

### Cell culture

Mouse lung epithelial MLE-12 cells were obtained from Cell Bank of Chinese Academy of Sciences (Shanghai, China). Cells were cultured with 1640 medium (Invitrogen, CA, USA) containing 1% penicillin–streptomycin (Gibco, MD, USA) and 10% fetal bovine serum (FBS, Invitrogen, CA, USA). Cells were maintained in a humified cell incubator at 37°C, 5% CO2. For cell damage induction, LPS was applied at 0.5 μg/ml [27], the induction time was indicated in the figure legend of each experiment. Additionally, to investigate the role of MAPK1 on LPS-induced inflammatory injury, cells were treated with 10 μM of PD98059 (MCE, NH, USA), a MAPK1 inhibitor, for 24 h. Cell line authentication was performed by STR DNA profiling analysis.

### Cell transfections

Small interfering RNA (siRNA) targeting circ_0001679 (si-circ_0001679#1, si-circ_0001679#2 and si-circ_0001679#3), circ_0001679 overexpression vector (circ_0001679), the scrambled siRNA negative control (si-NC), miR-338-3p mimic, miR-338-3p inhibitor and miRNA negative control (miR-NC), MAPK1 overexpression vector (MAPK1) and negative control were purchased from GenePharma (Shanghai, China). Cells were harvested by 0.25% trypsin digestion, and seeded into 6-well plate at the density of 3 × 105 cells/well. When cell confluence reached approximately 70%, the cells were transfected with different plasmids, siRNA or miRNA mimic/inhibitor using Lipofectamine 2000 reagent (Invitrogen, MA, USA) [25]. 48 hours post transfection, cells were treated with LPS (0.5 μg/ml) for 24 hours before functional assays. The sequences of synthesized oligonucleotides were displayed as below. Si-circ_0001679#1; 5’-AGACGAGAAUCCUGAGAAACA-3’, Si-circ_0001679#2 5’-CAAAGAGAGUUCGAAUAAAGG-3’; Si-circ_0001679#3 5’-GCAAAGAGAGUUCGAAUAAAG-3’; miR-338-3p 5ʹ-UCCAGCAUCAGUGAUUUUGUUG-3ʹ; miR-338-3p inhibitor 5ʹ-CAACAAAAUCACUGAUGCUGGA-3ʹ;circ_0001679 5ʹ-CTGAGCATCTTCCGTTTG-3ʹ; MAPK1 5ʹ-AGAACATCATTGGCATCA-3ʹ.

### Apoptosis detection by flow cytometry

The detection of cell apoptosis was performed using the FITC Annexin V Apoptosis Detection Kit (Beyotime, Shanghai, China) according to the manufacturer’s instructions. MLE-12 cells in different treatment groups were harvested by 0.25% trypsin digestion and cell concentration was adjusted to 1 × 10^6^/ml. 1 μL Annexin V-FITC and 1 μL PI were added to 1 mL cell resuspension for 30-min incubation in the dark. Stained cells were washed twice with PBS and the percentage of apoptotic cells was detected by BD FACS CantoTM II Flow Cytometer (BD Biosciences) [28].

### Cell counting kit-8 (CCK-8) assay

MLE-12 cells in different treatment groups were harvested by 0.25% trypsin digestion and cells were seeded in a 96-well-plate at a density of 1500 cell/well. Cells were cultured in a humidified cell culture incubator for 0, 24, 48, 72 and 96 hours, respectively. 10 μL CCK8 reaction solution (Solarbio, Beijing, China) was added to the cell culture at indicated time point and incubated for 3 h in a humidified cell culture incubator. The light absorption value (OD value) was recorded at 450 nm wavelength on a Synergy H1 microplate reader (Beckman Coulter, CA, USA).

### Enzyme-linked immunosorbent assay (ELISA)

The culture supernatants of MLE-12 cells in different treatment groups were collected, followed by 15 min of centrifugation at 1 000 × g and 4°C. The supernatants of each sample were collected and 100 µL supernatant was used for ELISA according to the manufacturer’s instructions of the ELISA assay kit [29]. After the color development, the OD values were detected by a microplate reader (Beckman Coulter, CA, USA). All the ELISA assay kits were provided by Abcam (Cambridge, MA, USA).

### Target prediction and dual luciferase assay

Starbase bioinformatics software (https://starbase.sysu.edu.cn/) [30] was used for predicting the biding site between circ_0001679 and miR-338-3p. Then the wild type (WT) and mutated (MUT) binding site was cloned into PmirGLO vector expressing firefly luciferase respectively (Promega). Plasmid containing the Renilla luciferase gene (pRL-TK) was used as a control plasmid. The reporter plasmid and Renilla luciferase control plasmid were co-transfected into cells with either miR-338-3p mimic or miR-NC in a 12-well plate (1 × 10^5 cells/well) using Lipofectamine 3000 reagent (Invitrogen, L3000001). The relative luciferase activity was recorded 48 h after transfection using the Dual-Luciferase Reporter Assay Kit (Promega, Mannheim, Germany) [25].

The predicted binding site between miR-338-3p and MAPK1 3’ untranslated region (3’UTR) was predicted using Starbase bioinformatics software. The wild type (WT) or the mutated (MUT) binding site between miR-338-3p and MAPK1 was cloned into PmirGLO luciferase reporter, respectively (Promega). Plasmid containing the Renilla luciferase gene (pRL-TK) was used as a control plasmid. The reporter plasmid and Renilla luciferase control plasmid were co-transfected into cells with either miR-338-3p mimic or miR-NC in a 12-well plate (1 × 10^5 cells/well) using Lipofectamine 3000 reagent (Invitrogen, L3000001). 48 h post transfection, the relative luciferase activities were measured using Dual-Luciferase Reporter Assay Kit (Promega). The relative firefly luciferase activity was normalized to that of Renilla luciferase in the control plasmid.

### Quantitative real-time polymerase chain reaction (qRT-PCR)

The MLE-12 cells in different treatment groups were collected, and Trizol reagent (Invitrogen) was utilized to extract total RNA. Based on manufacturer's instruction, 1 ml of Trizol reagent were added per 1 × 10^6^ cells. TaqMan miRNA reverse transcription kit (Applied Biosystems, CA, USA) or Takara reverse transcription kit (Takara, Dalian, China) was utilized for cDNA synthesis, while TaqMan miRNA quantitative PCR kit (Thermo Fisher Scientific, MA, USA) or real-time quantitative PCR kit (Takara, Dalian, China) was adopted for amplification on ABI 7500 RT-PCR System (Applied Biosystems Inc., CA, USA). β-actin was used as the reference for cicrRNA, and U6 was used as the reference for the moralization of miRNA expression. The 2-^ΔΔCt^ method was applied to calculate the relative expression of circ_0001679 and miR-338-3p [31]. The PCR cycling conditions used: 95°C for 30s for initial denaturation, and then 40 cycles of denaturation at 94°C for 30s, elongation at 60°C for 20s. In this study, all primers were synthesized by Shanghai Sangon Biotechnology Co., Ltd. (Shanghai, China): miR-338-3p sense 5’-GCAGTCCAGCATCAGTG-3’, anti-sense 5ʹ-CAGTGCGTGTCGTGGAGT-3’; U6 sense 5’-TCCGACGCCGCCATCTCTA-3’, antisense 5’-TATCGCACATTAAGCCTCTA-3’ circ_0001679 sense 5ʹ-CTGGACCCTGAGGATGCT-3’, anti-sense 5ʹ-ATGACCCTGCTTTGTGCA-3’; β-actin sense 5’-GGCTGTATTCCCCTCCATCG-3’, anti-sense 5’-CCAGTTGGTAACAATGCCATGT-3’.

### RNA pulldown assay

Biotin-labeled miR-NC (negative control) or Biotin-labeled miR-338-3p probe were synthesized by Ruiyuan Biological Company (Nanjing, China). Cells lysates were collected by IP lysis buffer (Beyotime, P0013) and were incubated with biotinylated miR-NC or Biotin-labeled miR-338-3p for 2 h. 10% of total cell lysates was saved as the input. The mixture was further incubated with M-280 streptavidin magnetic beads (Sigma-Aldrich) at 4°C for 4 h. A magnetic bar was used to precipitate the magnetic beads and associated nucleic acids, then the samples were washed 4 times with IP lysis buffer. Both the input and the pull-down samples were purified with Trizol reagent (Invitrogen) according to the manufacturer’s protocol. Reverse transcription and qPCR were performed to determine the relative expression of circ_0001679 in the pull-down samples [32].

### Western blotting (WB) assay

The procedures of Western blot were adopted from a previous study [33]. RIPA lysate was utilized for extracting total proteins from cells. Cells suspended in RIPA buffer were lyzed on ice for 10 mins and lysed cells were centrifuged at 14,000 rpm for 10 mins. The supernatant containing total protein lysate was quantified by a BCA Protein assay kit (Beyotime Biotechnology P0009; Shanghai, China). Aliquots of proteins (30 µg) were seperated through 10% SDS-PAGE, followed by the transfer of proteins onto PVDF membranes. After blocking with 5% skimmed milk for 1 hour, the membrane was then incubated with primary antibodies overnight at 4°C.: B-cell lymphoma-2 (Bcl-2) (1:500; ab196495, Abcam, Cambridge, UK), BCL2-associated x protein (Bax) (1:1000; ab32053, Abcam), Cleaved caspase 3 (1:1000; ab231289, Abcam), MAPK1 (1:800; ab265600, Abcam) and β-actin (1:5000; ab179467, Abcam). The membrane was washed 3 times with TBST for 5 minutes each. After wash, the membrane was further incubated with goat anti-rabbit IgG H&L (Abcam; ab96899, 1:5000) for additional 1 h under ambient temperature. Enhanced chemiluminescence ECL reagent (Pierce, IL, USA) was employed to visualize protein bands. The protein bands image was captured using Gel Doc 100 system and the Quantity One® imaging software (Bio-Rad, CA, USA).

### Statistical methods

SPSS21.0 (Chicago, IL, USA) was adopted for statistical analysis. Measurement data were presented as mean ± SD. The statistical difference between two groups was compared using unpaired Student’s t tests. Comparisons among multiple groups were analyzed using one-way analysis of variance (ANOVA) with Bonferroni post-hoc test. P < 0.05 is considered as statistically significant, each experimental result is the summary of at least three independent experiments.

## Results

In the present study, we aim to investigate the role of circ_0001679 in regulating the LPS-induced lung cell injury. We initially established an *in vitro* lung cell injury model using LPS-treated MLE-12 cells. siRNAs targeting circRNA_0001679 were employed to stably knock down circRNA_0001679, followed by functional assays to investigate the effect of circRNA_0001679 silencing. The levels of inflammatory cytokines such as IL-6, IL-β and TNF-α were detected by ELISA assay. We found that Circ_0001679 was upregulated in LPS-induced MLE-12 cells, and silencing circ_0001679 attenuated the growth inhibition and suppressed apoptosis induced by LPS. We further demonstrated that circ_0001679 functioned as a sponge of miR-338-3p to negatively regulate miR-338-3p activity. The upregulation of circ_0001679 maintained a high-level expression of MAPK1 by suppressing miR-338-3p. These data indicate that circ_0001679/miR-338-3p/MAPK1 axis may play an important role in acute lung injury.

### Knockdown of circ_0001679 ameliorates LPS-induced cell injury in MLE-12

**Figure 1.**
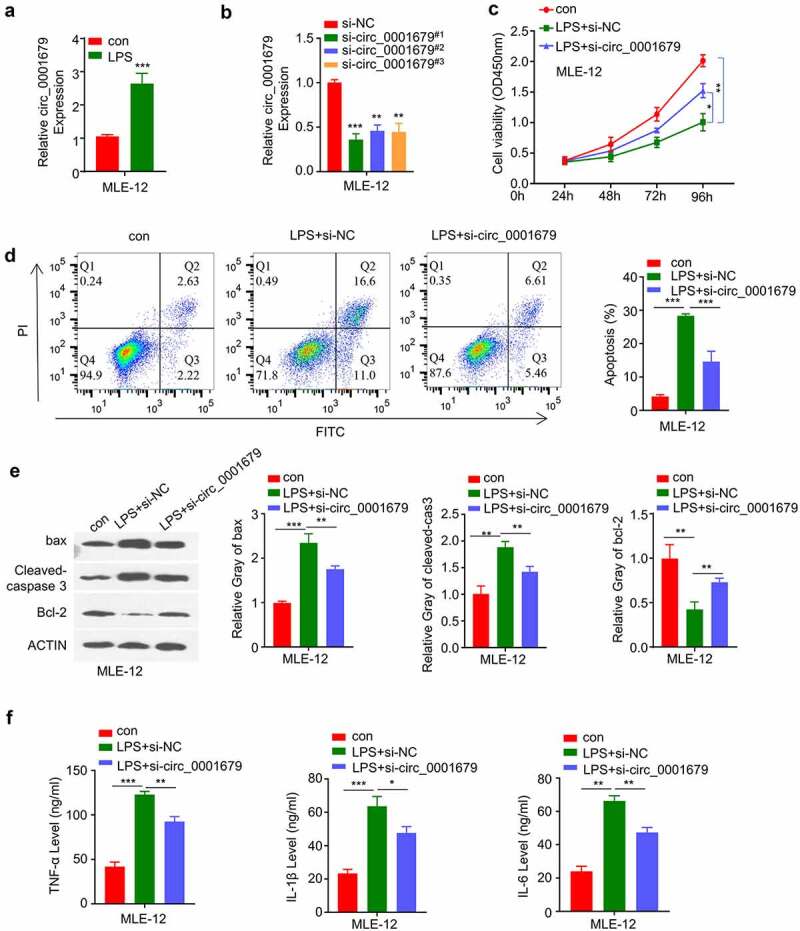
Knockdown of circ_0001679 inhibits LPS-induced cell injury in MLE-12. (a) Circ_00016791 expression was detected by qRT-PCR in MLE-12 cells after LPS treatment; (b) Circ_00016791 level was measured through qRT-PCR in MLE-12 cells after the transfection with siRNAs (NC, si-circ_0001679#1,#2,#3); (c) The light absorption at 450 nm in different groups of MLE-12 cells at 0 h, 24 h, 48 h and 72 h was detected by CCK-8; (d) The apoptosis levels in different MLE-12 cell groups were measured through flow cytometric analysis; (e) Apoptosis-related proteins Bcl2, cleaved-cas3, and Bax were measured in different groups of MLE-12 cells by Western blot; (f) IL-6, TNF-α, and IL-1β levels in different treatment groups were measured through ELISA. *p < 0.05; **p < 0.01; ***p < 0.001.

A previous study found that circ_0001679 was highly expressed in an LPS-induced ALI mice model, and silencing circ_0001679 could reduce sepsis-induced ALI injury [21]. In our study, we first examined the expression levels of circ_00016791 in MLE-12 upon LPS treatment. Circ_0001679 expression was significantly upregulated in MLE-12 cells after LPS induction (Figure 1a). We next silenced circ_00016791 expression in MLE-12 cell using siRNAs (si-circ_0001679#1, #2, #3). All three siRNAs significantly reduced the level of circ_00016791 in MLE-12 cells (Figure 1b), si-circ_0001679#1 showed a relatively higher knockdown efficiency and was used for the following study.

We next performed CCK-8 assay to investigate the effect of circ_00016791 in LPS induced cell proliferation. LPS treatment significantly suppressed cell proliferation in MLE-12 cells, while si-circ_0001679 transfection could partially rescue this effect (Figure 1c). Apoptosis detection by flow cytometry further revealed that LPS induced apoptotic events could also be partially suppressed by circ_0001679 silencing (Figure 1d). Consistently, LPS treatment reduced antiapoptotic protein Bcl-2 level and increased the levels of cleaved-cas3 and Bax. The transfection of si-circ_0001679 attenuated these changes (Figure 1e). We also analyzed the inflammatory cytokines by ELISA and we found that silencing circ_0001679 could mitigate the production of IL-6, IL-1β and TNF-α induced by LPS treatment (Figure 1f). Together, these results indicate that circ_0001679 upregulation is implicated in LPS-induced cell damages.

### Circ_0001679 overexpression alone does not cause cell injury in MLE-12

**Figure 2.**
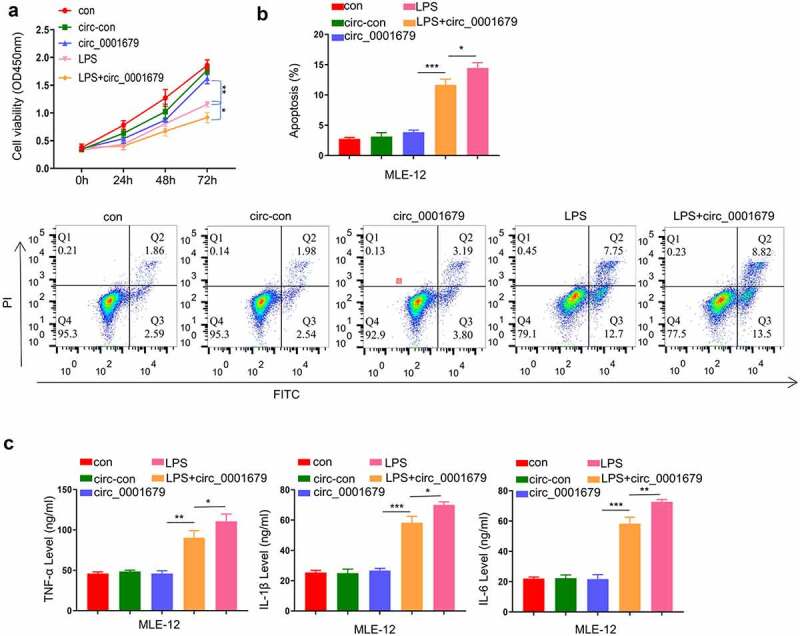
Circ_0001679 overexpression alone does not induce cell injury. (a) The light absorption at 450 nm in different groups of MLE-12 cells at 0 h, 24 h, 48 h and 72 h was detected by CCK-8; (b) The apoptosis levels in different MLE-12 cell groups were measured through flow cytometric analysis; (c) IL-6, TNF-α, and IL-1β levels in different treatment groups were measured through ELISA. *p < 0.05; **p < 0.01; ***p < 0.001.

To further confirm the regulatory role of circ_0001679 in LPS-induced cell injury, we constructed circ_0001679 overexpression vector and investigated the effect of circ_0001679 overexpression in cells with or without LPS treatment. Circ_0001679 overexpression alone did not affect cell proliferation, while in LPS-induced cells Circ_0001679 overexpression exacerbated the impairment of cell growth by LPS (Figure 2a). We further examined the apoptosis in different treatment groups. Consistently, circ_0001679 overexpression alone did not induce apoptosis in MLE-12 cells, while the apoptotic events of LPS-induced cells were significantly increased by circ_0001679 overexpression (Figure 2b). The analysis of inflammatory cytokines revealed a similar scenario, in which circ_0001679 overexpression did not affect the levels of TNF-α, IL-1β and IL-6 without LPS induction. However, upon LPS treatment circ_0001679 overexpression promoted the production of TNF-α, IL-1β and IL-6 (Figure 2c). Therefore, circ_0001679 overexpression is implicated in regulating the cell responses only when LPS-induced damages are initiated.

### Circ_0001679 targets miR-338-3p

**Figure 3.**
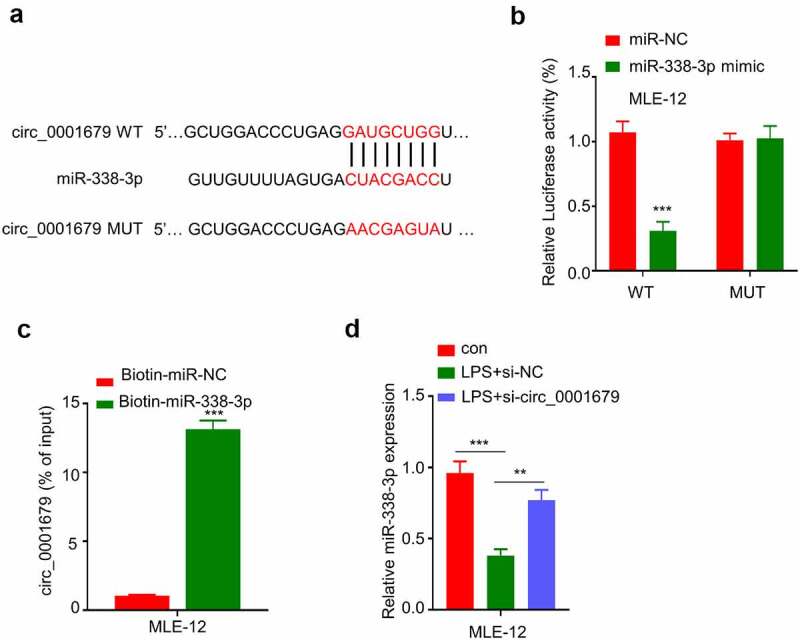
Circ_0001679 targets miR-338-3p. (a) Starbase was used to predict binding site between circ_00016791 with miR-338-3p; B-C, Functional interaction between circ_00016791 with miR-338-3p was confirmed by RNA pulldown assay (b) and luciferase reporter assay (c); (d) miR-338-3p expression in different treatment groups was measured through qRT-PCR. *p < 0.05; **p < 0.01; ***p < 0.001.

To the downstream mechanism by which circ_0001679 regulates LPS-induced cellular responses, we first predicted the downstream target miRNAs of circ_0001679 by starbase (http://starbase.sysu.edu.cn /index.php). We found that circ_0001679 contain many miRNA binding sites (Figure S1), and miR-338-3p was discovered to harbor a binding site for circ_0001679 (Figure 3a). We applied qRT-PCR to detect the expression of 7 target miRNAs which have been reported to be relevant in lung injury or LPS-induced inflammation response (The list of the literature was in supplementary file). Compared to LPS induced cells, silencing circ_0001679 could greatly upregulate the expression of miR-338-3p while the expression levels of other miRNAs were only mildly increased (Figure S2).Then we performed luciferase reporter assay using WT reporter or reporter containing mutated binding site between circ_00016791 with miR-338-3p. The presence of miR-338-3p mimic significantly inhibited luciferase activity in MLE-12 cells, while this effect was not observed in mutated reporter (Figure 3b). To further show the physical interaction between circ_00016791 with miR-338-3p, we performed RNA pull-down assay using biotin-labeled miR-338-3p probe. As compared to the miR-NC probe, miR-338-3p probe strongly enriched more circ_00016791 (Figure 3c). In addition, LPS treatment caused a downregulation of miR-338-3p, which could be largely reversed by the knockdown of si-circ_0001679 (Figure 3d). Together, these results suggest that circ_0001679 negatively target miR-338-3p.

### miR-338-3p mediates the effect of circ_0001679 in LPS-induced cell injury

**Figure 4.**
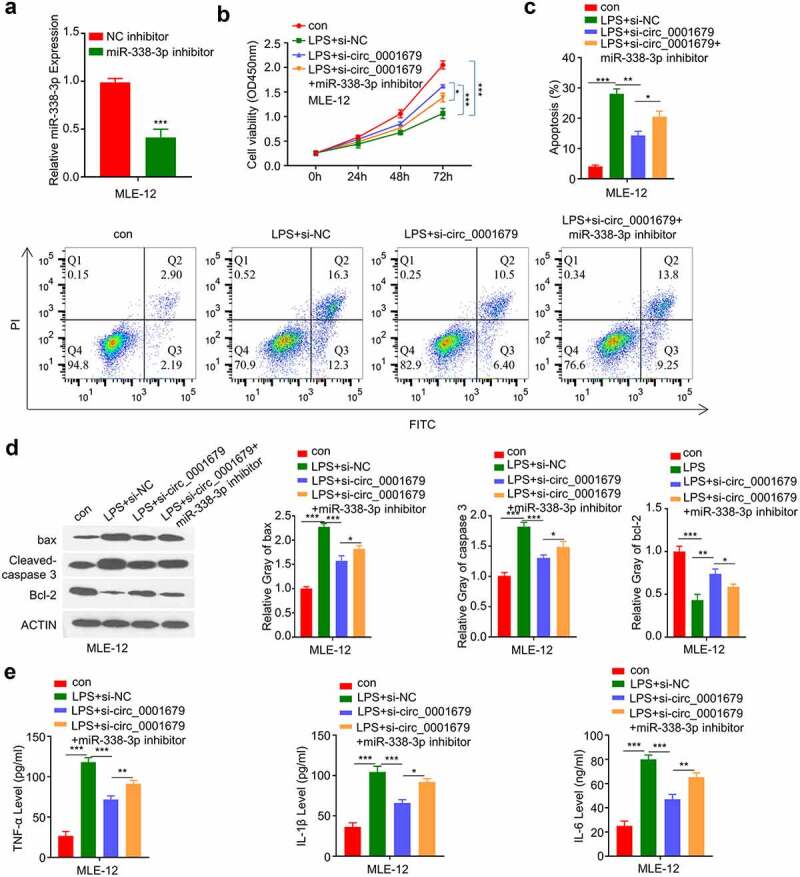
Knockdown of circ_0001679 inhibits LPS-induced cell injury in MLE-12 cells via regulating miR-338-3p. (a) miR-338-3p expression in different treatment groups was measured through qRT-PCR; (b) The light absorption value at 450 nm in different groups of MLE-12 cells at 0 h, 24 h, 48 h, 72 h were detected by CCK-8; (c) The apoptosis levels in different MLE-12 cell groups were measured through flow cytometric analysis; (d) The protein expression levels of Bcl2, cleaved-cas3, Bax and GAPDH in different experimental groups of MLE-12 cells were measured by Western blot; E, IL-6, TNF-α, and IL-1β levels in different treatment groups were measured through ELISA. *p < 0.05; **p < 0.01; ***p < 0.001.

We next sought to investigate whether miR-338-3p mediates the effect of circ_0001679 in LPS-induced cell damages. We applied miR-338-3p inhibitor which could markedly decrease the expression of miR-338-3p (Figure 4a). We found that the rescue effect of circ_00016791 silencing on LPS-induced cell damages were significantly impaired by miR-338-3p inhibitor. (Figure 4b). miR-338-3p inhibitor also impaired the protective effect of circ_00016791 silencing on LPS-induced apoptosis (Figure 4c). Consistently, si-circ_00016791 cause the increased of Bcl2 protein and the decrease of cleaved-cas3 and Bax levels after LPS induction, and miR-338-3p inhibitor partially reversed these effects (Figure 4d). Finally, we demonstrated that the rescue effect of si-circ_00016791 on the proinflammatory cytokine production (IL-6, TNF-α and IL-1β) was dampened by miR-338-3p inhibitor (Figure 4e).

### miR-338-3p targets MAPK1

**Figure 5.**
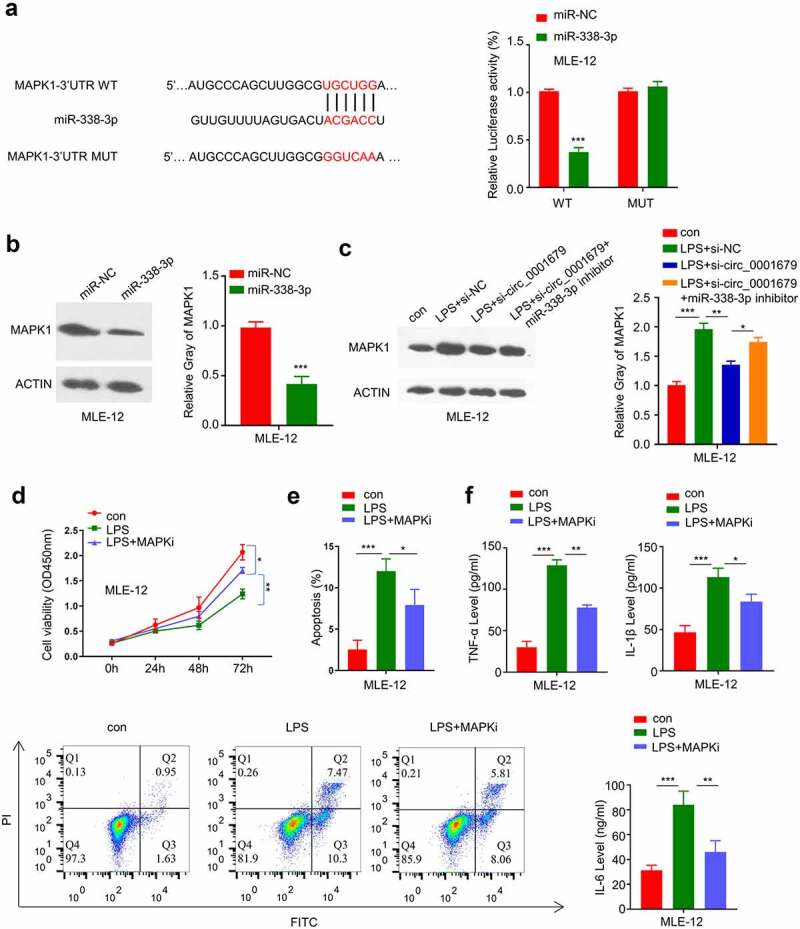
miR-338-3p targets MAPK1. (a) The presence of miR-338-3p binding site in the 3’ UTR of MAPK1 mRNA was predicted by starbase, and luciferase reporter assay was carried out to validate the functional interaction between miR-338-3p and MAPK1 mRNA; (b) MAPK1 protein expression was measured by Western blot; (c) MAPK1 protein expression in different treatment groups was measured by Western blot; (d) The light absorption at 450 nm in different groups of MLE-12 cells at 0 h, 24 h, 48 h and 72 h was detected by CCK-8; (e) The apoptosis levels in different MLE-12 cell groups were measured through flow cytometric analysis; (f) IL-6, TNF-α, and IL-1β levels in different treatment groups were measured through ELISA. *p < 0.05; **p < 0.01; ***p < 0.001.

The above results showed that knockdown of circ_0001679 inhibits LPS-induced MLE-12 cell injury through targeting miR-338-3p. We next searched for the downstream target mRNA of miR-338-3p, and found the presence of the miR-338-3p binding site in the 3ʹUTR of MAPK1 mRNA. Dual-luciferase reporter assay confirmed that miR-338-3p mimic was able to inhibit the luciferase activity of WT reporter containing the binding site between MAPK1 mRNA and miR-338-3p, while the activity of the MUT reporter containing the mutated binding site was not affected (Figure 5a). miR-338-3p overexpression markedly decreased MAPK1 protein level (Figure 5b). Moreover, we found that MAPK1 protein level was increased after LPS treatment and the upregulation was attenuated by si-circ_00016791; whereas miR-338-3p inhibitor co-transfection impaired the effect of si-circ_00016791 (Figure 5c).

To confirm a functional role of MAPK1 in LPS-induced cell damages, we applied an MAPK1 inhibitor PD98059 to investigate whether MAPK1 activity inhibition could rescue the effect of LPS-induced cell damages. MAPK1 inhibition could at least partially recue the LPS-induced growth inhibition (Figure 5d) and as well as decrease the apoptotic events (Figure 5e). Meanwhile, MAPK1 inhibition attenuated the increase of TNF -α, IL-1β and IL-6 levels in LPS-induced MLE-12 cells (Figure 5f). These data suggest that MAPK1 is a downstream effector in LPS-induced cell damages.

### MiR-338-3p/MAPK1 axis is implicated in LPS-treated MLE-12 cell injury

**Figure 6.**
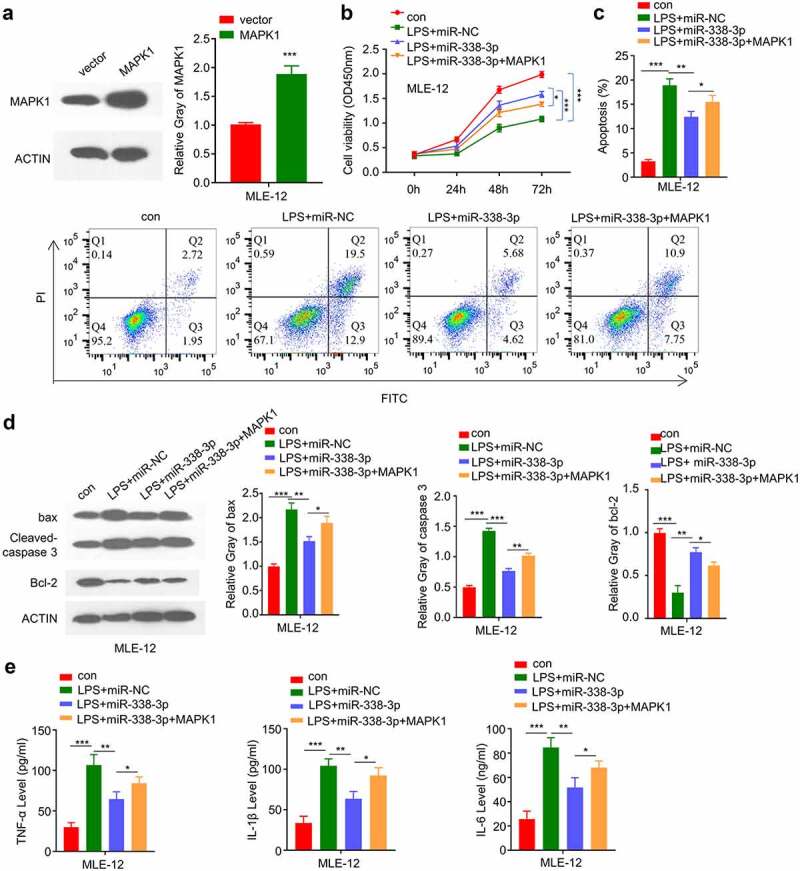
miR-338-3p inhibits LPS-induced cell injury by negatively regulating MAPK1 expression. (a) MAPK1 protein levels in different treatment groups were measured by Western blot; (b) The light absorption value at 450 nm at 0 h, 24 h, 48 h, 72 h in different groups of MLE-12 cells was detected by CCK-8; (c) The apoptosis levels in different MLE-12 cell groups were measured through flow cytometric assay; (d) The protein expression levels of Bcl2, cleaved-cas3, Bax and GAPDH in different groups of MLE-12 cells were measured by Western blot; (e) IL-6, TNF-α, and IL-1β expression levels in different treatment groups were measured through ELISA. *p < 0.05; **p < 0.01; ***p < 0.001.

We next attempted to validate whether miR-338-3p/MAPK1 axis is implicated in LPS-treated MLE-12 cell injury. We constructed an MAPK1 overexpression vector that could significantly promote the expression level of MAPK1 (Figure 6a). We then treated the cells with LPS, LPS+miR-338-3p mimic or LPS+miR-338-3p mimic+ MAPK1 overexpression. The cell growth inhibition by LPS was partially released by miR-338-3p mimic, and MAPK1 overexpression further impaired the effect (Figure 6b). MAPK1 overexpression also impaired the recue effect of miR-338-3p mimic on apoptosis (Figure 6c), which was accompanied by the decreased Bcl2 level and increased levels of cleaved-cas3 and Bax after MAPK1 overexpression (Figure 6d). Finally, we demonstrated that the rescue effect of miR-338-3p mimic on the proinflammatory cytokine production (IL-6, TNF-α and IL-1β) was dampened by MAPK1 overexpression (Figure 6e). The above results indicate that MiR-338-3p/MAPK1 axis is implicated in LPS-treated MLE-12 cell injury.

## Discussion

Although great achievements have been made in the diagnosis and treatment of ALI/ARDS, patients with ALI are still at a high mortality rate [34]. ALI/ARDS is characterized by a severe acute inflammatory response in the lungs, which can result in injuries of alveolar epithelial cells and pulmonary capillary endothelial cells [35]. Alveolar epithelial cells are the major components in the alveolar structure [36], which functions as a mechanical barrier for protecting against pulmonary damage, regulating pulmonary immune response and maintaining pulmonary fluid homeostasis [36,37].

A previous study reported that circ_0001679 was highly expressed in an LPS-induced ALI mice model, and silencing circ_0001679 could reduce sepsis-induced ALI injury [21]. Teng et al. [38] performed whole transcriptome analysis to show that circ_0001679 was highly expressed in lung tissues of the mice model of ALI. These studies strongly suggest that circ_0001679 upregulation is implicated in regulating the inflammatory damages in ALI. However, how circ_0001679 regulates the pathogenesis of ALI remains unclear. In the present study, we used an *in vitro* ALI model of LPS-induced cell damage in MEL-12 cell to study the functional role down the underlying mechanisms of circ_0001679. We demonstrated that circ_0001679 knockdown could prevent apoptosis and inflammatory response induced by LPS treatment. These data suggest that the knockdown of circ_0001679 can attenuate the inflammatory response in sepsis-induced ALI.

CircRNAs are able to modulate mRNA expression through sponging miRNAs [39]. In sepsis-related studies, it was found that circRNA_MA21 can ameliorate sepsis-induced renal injury by modulating the miR-9-3p/SMG1 axis [40]. circDMNT3B increases intestinal mucosal permeability in septic rats by regulating miR-20b-5p [41]; circC3P1 decreases proinflammatory cytokine production and apoptosis by regulating miR-21 in sepsis-induced ALI [42]. In the present study, we found that circ_0001679 sponges miR-338-3p, and circ00001679 negatively regulate miR-338-3p expression. Importantly, miR-338-3p inhibitor could dampen the effect of circ00001679 silencing in LPS induced cell model. These data altogether suggest miR-338-3p mediates the downstream effect of circ00001679. miR-338-3p is associated with inflammatory response in different disease models [26,43,44]. As demonstrated by Zhang et al., miRNA-338-3p could inhibit the inflammatory response in acetaminophen-induced acute liver injury through CAMK IIα signaling pathway [43]. In a mouse model of allergic encephalomyelitis, circRNA_001076/ miR-338-3p was found to promote immune inflammation via TRIM33/RORγt signaling pathway [44]. MiR-338-3p is reported to be sponged by circ_003867 in LPS-induced inflammatory injury model of human bronchial epithelial cell [26]. However, the expression pattern of miR-338-3p has not been reported in ALI patients. In our study, it was found that LPS treatment reduce the expression of miR-338-3p, which is mediated by the upregulation of circ_0001679. We further demonstrated that suppressing the expression of miR-338-3p could partially reverse the effect of circ_0001679 silencing on cell proliferation, apoptosis and inflammatory responses. Our data suggest that Circ_0001679/miR-338-3p axis is implicated in the regulation of LPS-induced ALI.

CircRNAs are able to regulate gene expression at post-transcriptional or transcriptional levels [45,46]. We further revealed that miR-338-3p directly target MAPK1 mRNA at 3ʹUTR and negatively regulate its expression. Mitogen-activated protein kinases (MAPKs), also known as extracellular signal-regulated kinase (ERK), participate in numerous biological processes, like cell differentiation, growth and cell cycle progression [47]. MAPK1 is a downstream target of MAPK, which is also known as p42 MAPK or ERK2 [48]. It can be activated by different physical or chemical stimuli, which in turn activates the P38 signaling pathway to regulate the immune responses [49]. It was found that in macrophages, the activation of MAPK1 pathway contributes to the enhanced inflammatory cytokine production, like IL-1, IL-6, and TNF-α [50]. In liver cirrhosis and inflammation, the degree of liver fibrosis and inflammation were correlated with MAPK1 activation [51]. In a LPS-induced ALI model, MAPK1 was reported to be regulated by miR-342 [52]. In current study, we found that MAPK1 inhibitor PD08059 was able to significantly ameliorate cell injury caused by LPS induction. In addition, MAPK1 overexpression mitigates effect of miR-338-3p mimic on LPS-induced MEL-12 injury. Overall our data suggest that circ_0001679/miR-338-3p/MAPK1 axis involved in regulating the inflammatory response in ALI.

However, many cellular factors can contribute to the acute lung injury caused by LPS. We found that knockdown of circ_0001697 could attenuate cell injury and inflammatory response induced by LPS, but could not completely inhibit these effects. This may be because that high expression of circ_0001697 is not the only underlying factor leading to cellular injury upon LPS treatment. In addition, targeting the miR-338-3p/MAPK1 axis could only attenuate the observed cellular effects caused by LPS treatment, but not prevent the cellular effects. We therefore conclude that targeting circ_0001697/miR-338-3p/MAPK1 axis may attenuate the pathogenesis of ALI, but could not cure the disease.

Our study provides a possible intervention target for the amelioration of clinical lung injury. However, more evidence is required to validate the role of circ_0001679/miR-338-3p/MAPK1 in human samples. Clinical samples from human ALI patients are required to validate the upregulation circ_0001679 and downregulation of miR-338-3p in human subjects. Meanwhile, the association of expression level of circ_0001679/miR-338-3p and the inflammatory conditions in ALI patients should be evaluated to inform whether circ_0001679/miR-338-3p axis can indicate the inflammatory conditions in patients. The potential protective effect of MAPK1 inhibitor in ameliorating LPS-induced cell damage should be examined in the ALI mouse model.

## Conclusion

In summary, our work demonstrated the upregulation of circRNA_0001679 in LPS-induced MLE-12 cells, and its silencing can alleviate LPS-induced cell injury through the miR-338-3p/MAPK1 axis. Our study suggests that circ_0001679 may serve as a potential target in ameliorating ALI, and the functional roles of circ_0001679/miR-338-3p/MAPK1 axis in ALI need to be further validated in human samples and animal models.

## Supplementary Material

Supplemental Material

## Data Availability

The data is available from the corresponding author on reasonable request.
