# Supplementary material for: Knockdown of circ_0001679 alleviates lipopolysaccharide-induced MLE-12 lung cell injury by regulating the miR-338-3p/ mitogen-activated protein kinase 1 axis
Source: Bioengineered. 2022 Mar 10;13(3):5803–17. doi: 10.1080/21655979.2022.2034564 (PMC8973724; doi:10.1080/21655979.2022.2034564)
Supplement: Supplemental Material [file KBIE_A_2034564_SM6132.zip › supplementary/supplementray file.docx]

**miR-423-5p**

Singh KP, Maremanda KP, Li D, Rahman I. Exosomal microRNAs are novel circulating biomarkers in cigarette, waterpipe smokers, E-cigarette users and dual smokers. BMC Med Genomics. 2020 Sep 10;13(1):128. doi: 10.1186/s12920-020-00748-3. PMID: 32912198; PMCID: PMC7488025.

**Primers:** >mmu-miR-423-5p MIMAT0004825

UGAGGGGCAGAGAGCGAGACUUU

F CAGTGAGGGGCAGAGAG

R GGTCCAGTTTTTTTTTTTTTTTAAAGTC

**miR-429 miR-200b-5p**

Xiao J, Tang J, Chen Q, Tang D, Liu M, Luo M, Wang Y, Wang J, Zhao Z, Tang C, Wang D, Mo Z. miR-429 regulates alveolar macrophage inflammatory cytokine production and is involved in LPS-induced acute lung injury. Biochem J. 2015 Oct 15;471(2):281-91. doi: 10.1042/BJ20131510. Epub 2015 Aug 20. PMID: 26431850.

**Primers:**

**miR-429-5p** >mmu-miR-429-5p MIMAT0017178

GUCUUACCAGACAUGGUUAGA

F CGCAGGTCTTACCAGACA

R GGTCCAGTTTTTTTTTTTTTTTCTAAC

**miR-200b-5p** >mmu-miR-200b-5p MIMAT0004545

CAUCUUACUGGGCAGCAUUGGA

F GCATCTTACTGGGCAGCA

R GGTCCAGTTTTTTTTTTTTTTTCCAA

**miR-541-5p miR-3065-3p**

Lee W, Kim I, Shin S, Park K, Yang K, Eun JW, Sul H, Jeong S. Expression profiling of microRNAs in lipopolysaccharide-induced acute lung injury after hypothermia treatment. Mol Cell Toxicol. 2016;12(3):243-253. doi: 10.1007/s13273-016-0029-7. Epub 2016 Oct 7. PMID: 32226458; PMCID: PMC7096978.

**Primers:**

>mmu-miR-541-5p MIMAT0003170

AAGGGAUUCUGAUGUUGGUCACACU

F GAAGGGATTCTGATGTTGGTC

R GTCCAGTTTTTTTTTTTTTTTAGTGTG

>mmu-miR-3065-3p MIMAT0014837

UCAGCACCAGGAUAUUGUUGGGG

F GCAGTCAGCACCAGGAT

R CAGTTTTTTTTTTTTTTTCCCCAAC

**miR-338-3p**

Liu G, Wan Q, Li J, Hu X, Gu X, Xu S. Circ_0038467 regulates lipopolysaccharide-induced inflammatory injury in human bronchial epithelial cells through sponging miR-338-3p. Thorac Cancer. 2020 May;11(5):1297-1308. doi: 10.1111/1759-7714.13397. Epub 2020 Mar 17. PMID: 32181994; PMCID: PMC7180556.

**Primers:**

>mmu-miR-338-3p MIMAT0000582

UCCAGCAUCAGUGAUUUUGUUG

F 5’-GCAGTCCAGCATCAGTG-3’,

R 5'-CAGTGCGTGTCGTGGAGT-3'

**miR-770-5p**

Yuan J, Li P, Pan H, Xu Q, Xu T, Li Y, Wei D, Mo Y, Zhang Q, Chen J, Ni C. miR-770-5p inhibits the activation of pulmonary fibroblasts and silica-induced pulmonary fibrosis through targeting TGFBR1. Ecotoxicol Environ Saf. 2021 Sep 1;220:112372. doi: 10.1016/j.ecoenv.2021.112372. Epub 2021 May 31. PMID: 34082245.

**Primers:**

>mmu-miR-770-5p MIMAT0004822

AGCACCACGUGUCUGGGCCACG

F AGAGCACCACGTGTCT

R GGTCCAGTTTTTTTTTTTTTTTCGT

**U6**

F 5’-TCCGACGCCGCCATCTCTA-3’,

R 5’-TATCGCACATTAAGCCTCTA-3’
