# Supplementary figures and images for: Knockdown of circ_0001679 alleviates lipopolysaccharide-induced MLE-12 lung cell injury by regulating the miR-338-3p/ mitogen-activated protein kinase 1 axis
Source: Bioengineered. 2022 Mar 10;13(3):5803–17. doi: 10.1080/21655979.2022.2034564 (PMC8973724; doi:10.1080/21655979.2022.2034564)

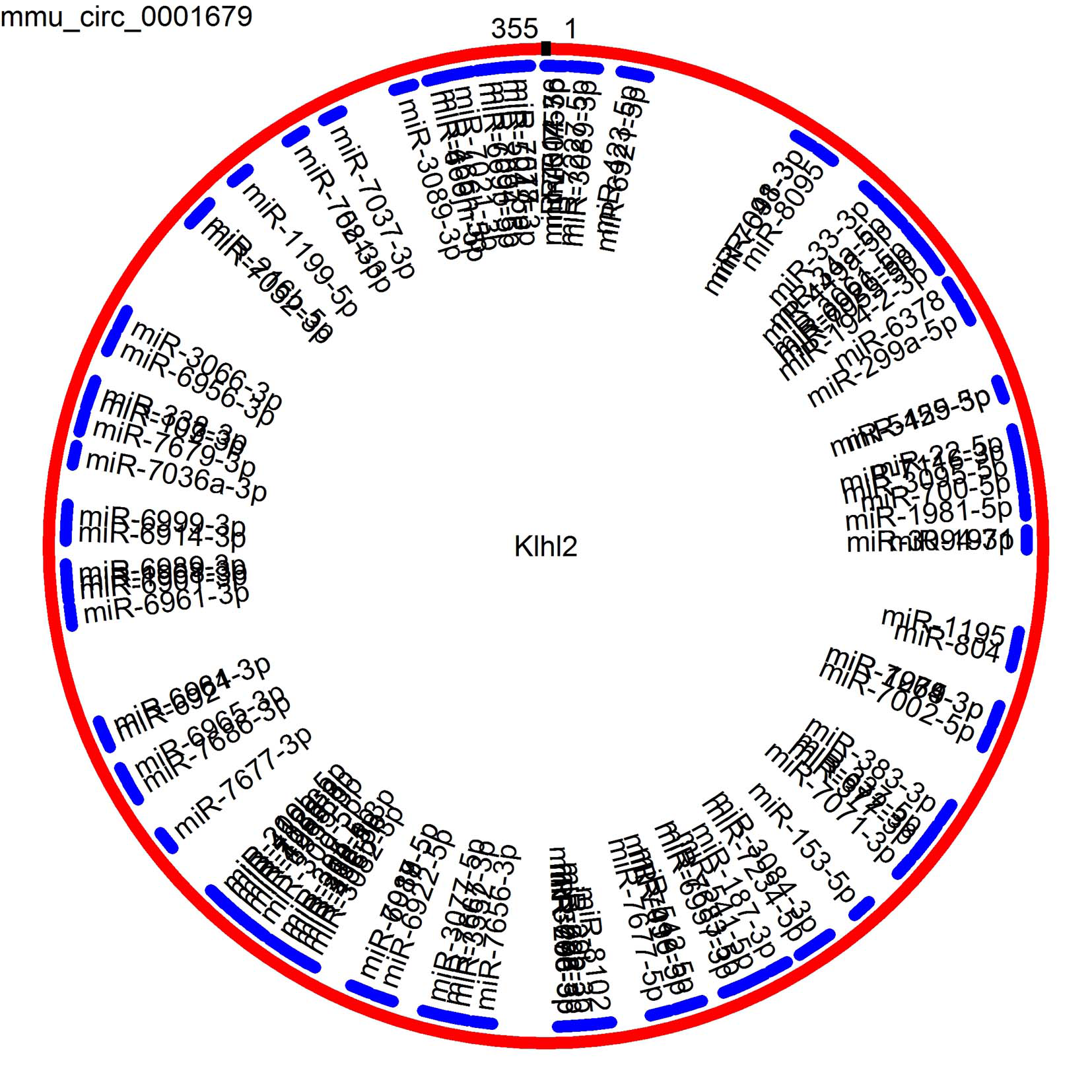

Supplement: Supplemental Material [file KBIE_A_2034564_SM6132.zip › supplementary/Figure S1.tif]

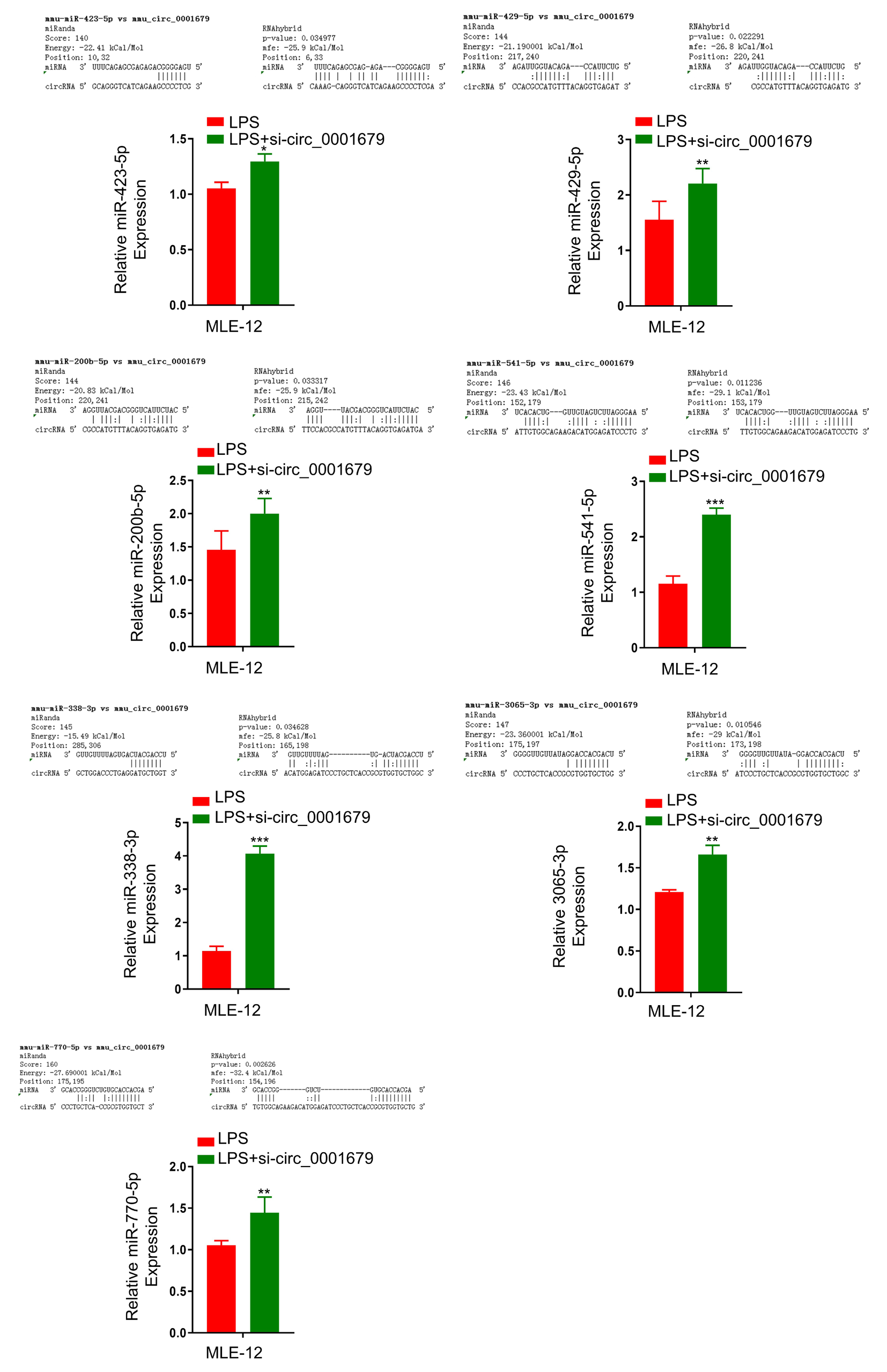

Supplement: Supplemental Material [file KBIE_A_2034564_SM6132.zip › supplementary/Figure S2.tif]
